# Effects of tamoxifen on the immune response phenotype in equine peripheral blood mononuclear cells

**DOI:** 10.3389/fvets.2024.1381162

**Published:** 2024-04-10

**Authors:** Maksimiano Rodríguez, John Quiroga, Bayron Cortés, Gabriel Morán, Claudio Henríquez

**Affiliations:** Instituto de Farmacología y Morfofisiología, Facultad de Ciencias Veterinarias, Universidad Austral de Chile, Valdivia, Chile

**Keywords:** tamoxifen, horse, T lymphocytes, immune response polarization, T regulatory cells, cytotoxicity

## Abstract

Tamoxifen (TAM) is widely utilized in the prevention and treatment of human breast cancer and has demonstrated the potential to modulate the immune response. It has been proposed as a therapeutic tool for immune-mediated diseases. TAM has been investigated as a possible treatment for asthma-like conditions in horses, revealing specific impacts on the innate immune system. While the effects of TAM on equine neutrophils are well-documented, its influence on lymphocytes and the modulation of the immune response polarization remains unclear. This *in vitro* study employed peripheral blood mononuclear cells (PBMC) from healthy horses, exposing them to varying concentrations of the TAM and assessing the expression of genes involved in the polarization of the immune response (*TBX21*, *IFNG*, *GATA3*, *IL4*, *IL10*, *FOXP3*, and *CTLA4*) in PBMC stimulated or not with PMA/ionomycin. Additionally, the effect of TAM over the proportion of regulatory T cells (Treg) was also assessed. TAM did not significantly affect the expression of these genes and Treg at low concentrations. However, at the highest concentration, there was an impact on the expression of *GATA3*, *IL4*, *IL10*, and *CTLA4* genes. These alterations in genes associated with a Th2 and regulatory response coincided with a noteworthy increase in drug-associated cytotoxicity but only at concentrations far beyond those achieved in pharmacological therapy. These findings suggest that the effects of TAM, as described in preclinical studies on asthmatic horses, may not be attributed to the modification of the adaptive response.

## 1 Introduction

Tamoxifen (TAM) is a selective estrogen receptor modulator (SERM) that belongs to the triphenylethylene group of molecules and is used as a treatment for all stages of estrogen-positive human breast cancer (1). TAM has also been shown to have therapeutic effects in different inflammatory pathologies in humans and animals. For instance, in patients suffering from corticosteroid-resistant Riedel’s disease, a rare chronic inflammatory pathology of the thyroid gland, a remission of the disease induced by TAM was reported (2). Similarly, successful treatment of a dermatomyositis rash resistant to conventional systemic immunosuppressants was reported using TAM (3). In a randomized, double-blind breast cancer prevention trial carried out on 111 healthy women with oral TAM treatment for 6 months, the drug was associated with significant reductions of C-reactive protein, fibrinogen, and cholesterol compared to placebo (4, 5). Moreover, Duffy et al. (6) suggest that TAM might help treat mast cell-mediated disease and other inflammatory diseases such as refractory asthma, pulmonary fibrosis, and mastocytosis, making it worthy of further clinical trials.

These findings about the anti-inflammatory effect of TAM described in humans correlate with *in vitro* trials and preclinical models. The activation of the nuclear factor kappa B (NF-κB) is one of the earliest events that mediate inflammation and suppression of apoptosis induced by various stimuli in most cells (7); the expression of most genes involved in inflammation -such as COX-2- or in cellular proliferation—such as cyclin D1- are regulated by NF-κB. In an *in vitro* comparative study of several non-steroidal anti-inflammatory drugs (NSAIDs), TAM had the highest inhibitory activity on NF-κB. The same study reported that TAM was two times better than dexamethasone and more than 500 times than aspirin regarding the inhibition of TNF-induced NF-κB activity (8). Other results indicate that preventive TAM treatment interferes with all aspects of the allergic immune response, leading to a reduction of allergen-specific Ig levels in mice, a skewing effect in the T cell compartment with the inhibition of IL-4 and an abrogation of ear swelling responses in dermatitis (9). Further evidence that supports an immunomodulatory activity of TAM *in vivo* includes mice developing autoimmune skin disease (10–13), murine experimental autoimmune encephalomyelitis (14), and experimental autoimmune uveitis in rats. In particular, in this last animal model, the concurrent intraocular injection of 17β-estradiol did not fully reverse the effects of TAM, suggesting that TAM modulates immune function partly through an estrogen-independent mechanism (15).

As in humans, TAM in horses has also shown potent anti-inflammatory effects, mainly by modulating certain neutrophilic functions. We have described that TAM can reduce neutrophil infiltration in the airways of animals with asthma exacerbation (16). An independent group also found that TAM improves airway resistance in horses with exacerbated severe equine asthma, although no significant improvement in BALF neutrophil counts was observed (17). Some of these effects can be explained due to the direct effect of this drug on equine neutrophils, which are the primary inflammatory cells in this equine asthma. For example, we have described that TAM can inhibit the respiratory burst (18, 19), potentially modulating tissue damage induced by this mechanism. Additionally, this drug can inhibit chemotaxis and chemokinesis (18, 20), potentially preventing the arrival of inflammatory cells. Another effect described is the induction of apoptosis in neutrophils, both *in vitro* and *in vivo* (16, 21), as well as an increase in the efferocytosis of these apoptotic cells (22), which can favor the resolution of the inflammatory process. All these results suggest that TAM can modulate neutrophilic functionality, which partly explains the beneficial effect of this drug in horses with airway inflammation. However, there is still no evidence of a role for TAM in cells of the horse adaptive immune system. Therefore, this study aims to evaluate the *in vitro* effect of TAM on genes related with the polarization of the adaptive immune response and regulatory T cells (Treg) population.

## 2 Materials and methods

### 2.1 Animals and blood sampling

Blood samples were obtained from five healthy mixed-breed adult horses (body weight 420–450 kg, ages 8 to 15), including both sexes. These horses belong to the Universidad Austral de Chile and were maintained at the Veterinary Teaching Hospital. All procedures were approved and performed by the contours of the Ethics and Bioethics committee from the Universidad Austral de Chile (form number 418/2021). Sampling consists of obtaining 18 mL of peripheral blood from each animal after jugular venipuncture, which was placed in sterile tubes containing 2 mL of 3.8% trisodium citrate.

### 2.2 Cell isolation, culture, and stimulation

Peripheral blood mononuclear cell (PMBC) isolation was performed according to the protocol described by Caffi et al. (23). Briefly, 2.5 mL of 80% (bottom) and 70% (top) Percoll^™^ (GE Healthcare, United States) were placed in 15 mL-conical tubes. Density solutions were achieved by diluting Percoll^™^ with PBS-citrate (8.1 mM Na_2_HPO_4_, 1.4 mM KH_2_PO_4_, 137 mM NaCl, 2.7 mM KCl, 15.4 mM C₆H₅Na₃O₇ × 2 H₂O, pH 7.4). A 2.5 mL-volume of whole blood was placed on both solutions. After centrifugation (20 min, 300 × g), PBMC were obtained by pipetting the upper layer, washed, and resuspended in PBS-citrate for count. After that, cells were cultured in 24 well plates at a concentration of 5 × 10^6^ cells/mL in 1 mL of RPMI with 10% fetal bovine serum (FBS) and penicillin (100 units/mL), streptomycin (0.1 mg/mL) and amphotericin B (0.25 μg/mL) at 37°C and 5% CO_2_ overnight. The following day, cells were treated with TAM (Sigma-Aldrich, Saint Louis, MO, United States) at 0.01, 0.1, 1, and 10 μM concentrations or vehicle (DMSO 0.1%) and stimulated or not with Phorbol 12-myristate 13-acetate (PMA; 20 ng/mL, Merck) and ionomycin (Io; 1 μM, Tocris). All groups were incubated for 6 h at 37°C and 5% CO_2_.

### 2.3 Gene expression assessment

The assessment of the effect of TAM in the expression of genes related to polarization of the immune response and regulatory mechanism was performed by RT-qPCR. Cells were recovered using cell scrappers (SPL Life Sciences, Gyeonggi-do, Korea), and RNA was isolated using TRIzol^™^ (Life Technologies, Carlsbad, CA, United States) following the provider’s instructions (Thermo Fisher Scientific). DNA was removed with the kit DNAse Turbo (Thermo Fisher Scientific) after which RNA was quantified using a NanoDrop Lite^™^ (Thermo Fisher Scientific). Complementary DNA (cDNA) was synthesized using at least 1 μg of RNA, Oligo (dT) Primer, and M-MLV Reverse transcriptase, (all reagents from Promega Corporation, Madison, WI, United States), and stored at −80°C until further use.

qRT-PCR assays were performed using SYBR^™^ Green qPCR Master Mix (Bio-Rad, Hercules, CA, United States) was used according to the parameters defined by the manufacturer, in a QuantStudio^™^ 3 Real Time PCR System (Thermo Fisher Scientific). Polarization of the adaptive immune response was determined by the expression of the genes *TBX21* and *IFNG* for the Th1 phenotype, *GATA3* and *IL4* for the Th2 phenotype, and *RORC* and *IL17* for the Th17 phenotype. *IL10*, *FOXP3*, *TGFB1*, and *CTLA4* expression were evaluated as regulatory genes, while *YWHAZ* was used as housekeeping. Specific primers are presented in Table 1. The calculation of the differential expression between conditions was carried out using the 2^–ΔΔCT^ method, according to Livak and Schmittgen (24).

**Table 1:** Sequence, efficiency, and product size of the primers used.

Gene	NCBI reference sequence	Phenotype	Primer sequence (5′–3′)	Efficiency	*R* ^2^	Slope	Product size (bp)
*TBX21*	XM_023652657.1	Th1	Fwd: CGGGGCTGGTACTTATGGAG	103.06%	0.998	−3.251	172
			Rev: GCAACATGCCAGGAAACCAC				
*IFNG*	NM_001081949.1	Th1	Fwd: CATGATTTATTTTGGACGTTTTGGA	99.32%	0.999	−3.338	167
			Rev: CGTCAGCTACATCTGAATGACT				
*GATA3*	XM_023631902.1, XM_014737148.2, XM_023631903.1, XM_023631904.1	Th2	Fwd: TGGAGGAGAAATGCCAACGG	109.81%	0.997	−3.107	156
			Rev: GGAGCTGCTCTTGGGAAAGT				
*IL4*	NM_001082519.1	Th2	Fwd: AGGTTTCCTGCGTCAAGATG	109.82%	0.999	−3.107	150
			Rev: GACATGGTGCCTTGAGGGAG				
*RORG*	XM_001916045.5, XM_014739881.2	Th17	Fwd: GGAAGTGGTGCTTGTCAGGA	UD	UD	UD	132
			Rev: TCGGAAAAGCGTAAGGCACT				
*IL17*	NM_001143792.1	Th17	Fwd: TGAGAACTTCATCCGTGTCACT	UD	UD	UD	109
			Rev: TTCTTGTCCCCAGTGTTCGG				
*IL10*	NM_001082490.1	Treg	Fwd: AGATGCTGCGCTTCTACACA	95.64%	0.998	−3.431	120
			Rev: TGAAGATGTCGAACTCCCCC				
*TGFB1*	NM_001081849.1	Treg	Fwd: GGAATGGCTGTCCTTTGATG	91.24%	0.996	−3.551	120
			Rev: CGGAGTGTGTTATCTTTGCTGTC				
*CTLA4*	XM_023622472.1, XM_023622473.1	Treg	Fwd: GGTCCGAGTGACAGTGCTAC	109.78%	0.999	−3.108	183
			Rev: CAGATGTAGAGCCCCGTGTC				
*PDCD1*	XM_023642813.1, XM_023642814.1, XM_023642815.1	Treg	Fwd: TTCCACATGAGCGTCCTCG	99.811%	0.995	−3.327	198
			Rev: CACGACCAGGCCCTGTAACT				
YWHAZ	XM_014728222.2, XM_001492988.6	HK	Fwd: TGTTCCCAACCATGTCCCAT	102.30%	0.999	−3.268	163
			Rev: GGGTCAAGAACATTGTGGCTG				

HK, housekeeping; Treg, regulatory T cells; Th, T helper; UD, undetermine.

### 2.4 Regulatory T cells determination by flow cytometry

In order to determine the effect of TAM over Treg cells, this cell subset was identified by flow cytometry as described by Henriquez et al. (25). Briefly, PBMCs were exposed to TAM (1 and 10 μM) or 0.1% DMSO, and incubated for 6 h at 37°C and 5% CO_2_. After that, cells were recovered, washed, counted, and placed in flow cytometry tubes (1 × 10^6^ cells per tube). For Treg staining, the following antibodies were used: mouse anti-horse CD4—FitC (dilution 1:10, clone CVS4; Thermo Fisher Scientific), goat anti-human CD25 (dilution 1.25:10, AF-223, R&D systems, Minneapolis, MN, United States), chicken anti-goat IgG (H + L)—Alexa Fluor 647 (dilution at 1:2,500, Invitrogen^™^, Thermo Fisher Scientific), all of which were incubated in 100 μL of flow cytometry staining buffer (FCSB, PBS + 1% FBS) for 1 h at room temperature (RT) in the dark. After incubation, cells were washed and permeabilized using FOXP3/Transcription Factor Staining Buffer Set (eBioscience, Thermo Fisher Scientific) according to the manufacturer’s instructions. For FoxP3 staining, cells were incubated in 100 μL of permeabilization buffer for 1 h with anti-mouse/rat FoxP3—PE antibody (dilution 2.5:100, clone FJK-16s, eBioscience, Thermo Fisher Scientific). Viable cells were identified by using LIVE/DEAD^™^ Fixable blue death cell stain kit according manufacturer instruction. Lastly, samples were suspended in 500 μL of FCSB and assessed by flow cytometry (BD FACSCanto^™^ II; Becton Dickinson, San Jose, CA, United States).

### 2.5 Cell cytotoxicity assessment

Cell cytotoxicity was assessed by measuring lactate dehydrogenase (LDH) activity using the CyQUANT^™^ LDH Cytotoxicity Assay—Fluorescence Kit (Invitrogen^™^) following the manufacturer’s instructions. Briefly, 2.5 × 10^5^/well PBMCs were cultured in a 96-well plate RPMI + 10% FBS overnight at 37°C and 5% CO_2_. The following day, each group was incubated for 6 h at 37°C and 5% CO_2_, with DMSO (0.1%), 1 or 10 μM of TX, with or without PMA 20 ng/mL + Io 1 μM. Cell conditions with only culture medium were used as controls for spontaneous and maximum release of LDH. All conditions were done in duplicate. After 5 h of incubation, 10 μL of H_2_O was added to the control condition, while the same volume of lysis buffer provided by the kit was added to the maximum LDH release condition. After 6 h of incubation, 50 μL of each condition was transferred to a new well, in which 25 μL of reporter mix + 25 μL of reagent mix was added. The samples were incubated in the dark at room temperature for 10 min, after which 50 μL of the stop solution was added. Fluorescence was measured using the Infinite M Nano + Tecan^™^ equipment (Männedorf, Switzerland) at 560 nm excitation and 590 nm emission. Cytotoxicity percentages were calculated according to the manufacturer’s instructions, considering the values for spontaneous and maximum LDH activity.

### 2.6 Statistical analysis

Data is shown as mean ± standard deviation (SD). Relative gene expression values were obtained with the software Desing & Analysis 2.6.9 (Thermo Fischer Scientific) and expressed as 2^–ΔΔCT^. After variance homoscedasticity analysis, comparisons between 3 or more groups were made using one-way ANOVA followed by Dunnett’s multiple comparison test or Kruskal–Wallis test followed by Dunn’s test, as appropriate. Values of *p* < 0.05 were considered significant. Statistical analyses were performed with PRISM v10.0 software (GraphPad, San Diego, CA, United States).

## 3 Results

### 3.1 Effect of TAM on immune response phenotype associated genes

**Figure 1:**
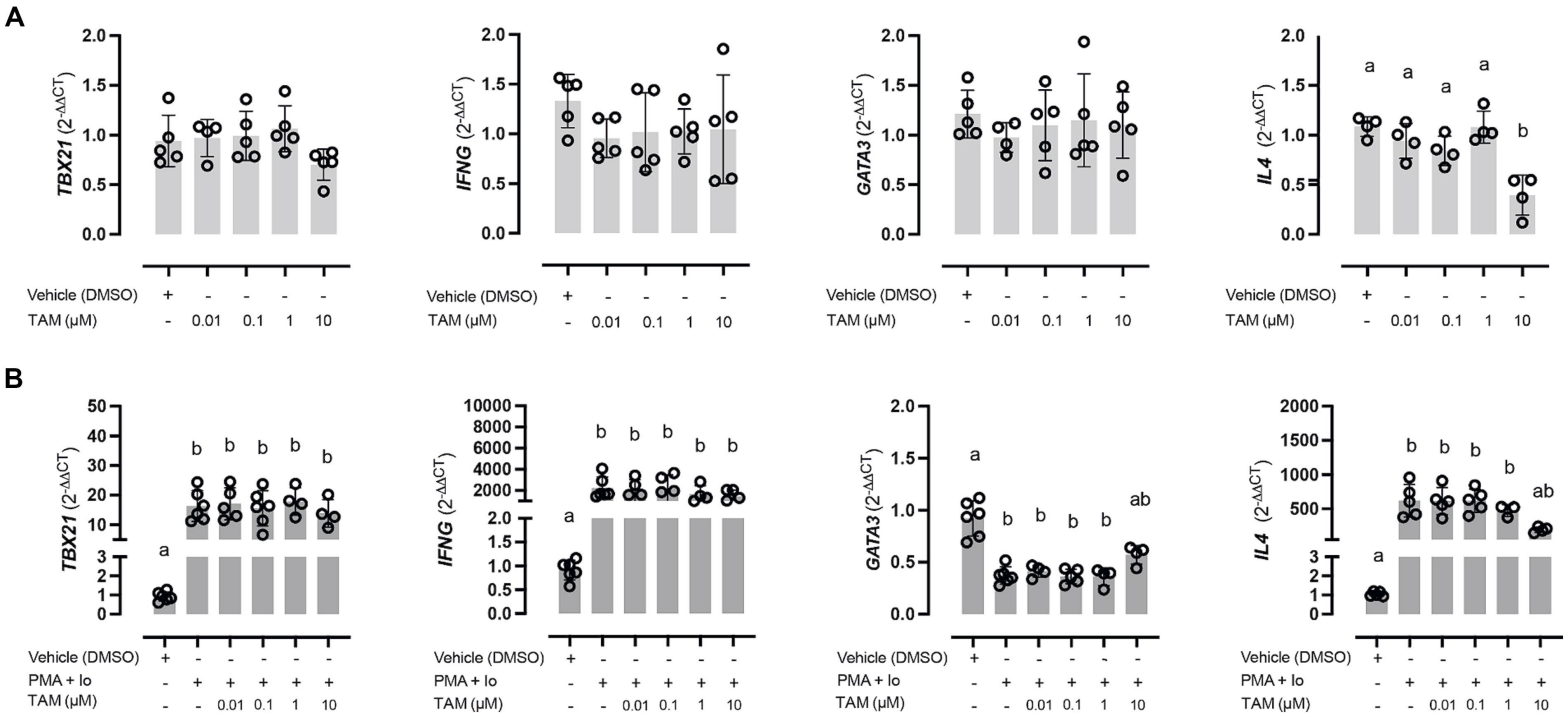
High concentrations of tamoxifen (TAM) modulate Th2-type immune response in equine PBMC. Expression of Th1 (*TBX21* and *IFNG*) and Th2 (*GATA3* and *IL4*) key genes in unstimulated **(A)** and PMA (20 ng/mL) + ionomycin (1 μM) stimulated **(B)** equine PBMC, incubated for 6 h with different concentrations (0.01–10 μM) of TAM of its vehicle (DMSO 0.01%). Different letters indicate statistical differences (*p* < 0.05), *n* = 4–5.

When evaluating the effect of incubating PBMCs with different concentrations of TAM, no significant changes were observed regarding the expression of the evaluated genes related to the polarization of the immune response (Figure 1A). The only gene that demonstrated a variation in its relative expression was *IL4*, decreasing significantly with the use of TAM highest concentration (10 μM) relative to the rest of the experimental conditions. However, this was not associated with a decrease in GATA3 expression despite what would have been expected. On the other hand, there were no significant variations in the expression of the genes associated with a Th1 response (*TBX21* and *IFNG*). Concerning the genes related to a Th17 response (*RORG* and *IL17*), it was impossible to evaluate their expression since their expression was very low and did not allow the amplification efficiency to be determined.

About the expression of genes associated with the immune response phenotype, specifically in response to stimulation versus the PMA/Io combination, all genes showed a modification in their expression. Genes of a Th1 type response, both *TBX21* and *IFNG*, significantly increased their expression (Figure 1B, left panels). TAM did not modify this increase in any of its concentrations. On the other hand, the genes associated with the Th2 response were shown to behave differently in the face of stimulation, observing a marked decrease in the expression of *GATA3*. In contrast, a significant increase was observed in the case of *IL4*. In both cases, this alteration was reversed in the condition incubated with the highest concentration of TAM (Figure 1B, right panels).

### 3.2 Effect of TAM on Treg cells

**Figure 2:**
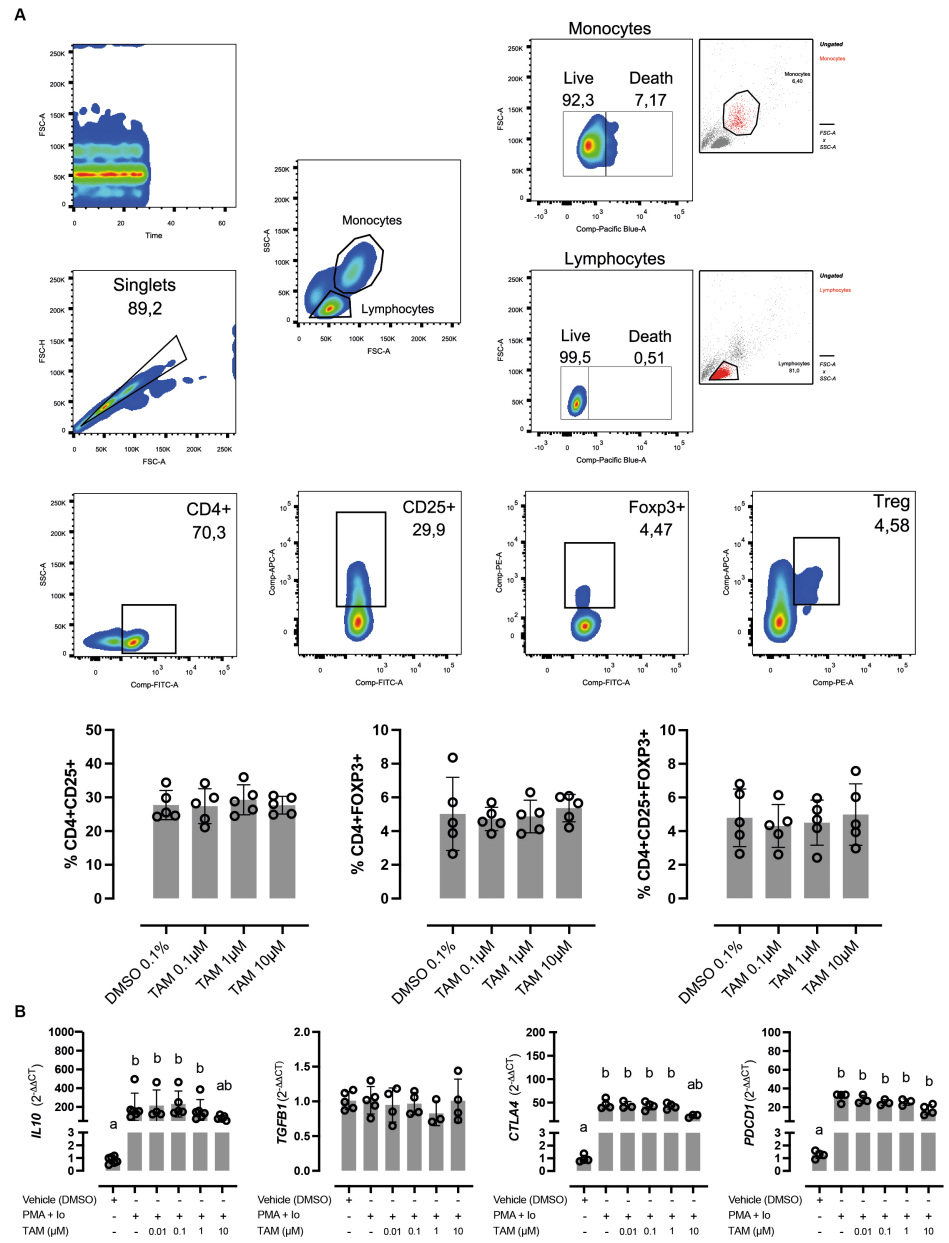
High concentrations of tamoxifen (TAM) modulate regulatory cytokines expression without affecting Treg proportion in equine PBMC. **(A)** In the upper panels, the gating strategy for the cellular subpopulation assessment is present. In lower panels, the effect of different concentrations (0.1–10 M) of TAM during 6 h on the proportion of CD4^+^ CD25^+^, CD4^+^ Foxp3^+^, and CD4^+^ CD25^+^ Foxp3^+^ cells on equine PBMC is shown. **(B)** Expression of regulatory genes (*IL10*, *TGFB1*, *CTLA4*, and *PDCD1*) in PMA (20 ng/mL) + ionomycin (1 μM) stimulated equine PBMC, incubated for 6 h with different concentrations (0.01–10 μM) of TAM of its vehicle (DMSO 0.01%). Different letters indicate statistical differences (*p* < 0.05), *n* = 4–5.

Due to the described effects of TAM as a modulator of the immune response, we were interested in evaluating whether this drug affects Treg cells, which represent one of the main subtypes of cells responsible for modulating the immune response in the periphery. In this regard, TAM did not induce changes *in vitro* in the proportion of CD4^+^, CD4^+^ CD25^+^, CD4^+^ FoxP3^+^, or CD4^+^ CD25^+^ FoxP3^+^ cells (Figure 2A). The effect of TAM on the expression of genes involved in regulating the response and that are commonly (although not exclusively) expressed by Tregs, such as *IL10*, *TGFB1*, *CTLA4*, and *PDCD1* was also evaluated (Figure 2B). Incubation for 6 h with TAM at different concentrations did not modify the expression of these genes (data not shown). On the other hand, stimulation of PBMCs with PMA/Io induced overexpression of *IL10*, *CTLA4*, and *PDCD1* but not *TGFB1*, an increase that was inhibited when incubated with TAM at its highest concentration (10 μM), similar to what occurred in the case of the associated genes with the Th2 response. The proportion of viable lymphocytes was greater than 99.1% in all conditions, while in the case of monocytes it varied between 92.6% in the vehicle condition and 90.8% TAM 10 μM.

### 3.3 Effect of TAM on cytotoxicity

**Figure 3:**
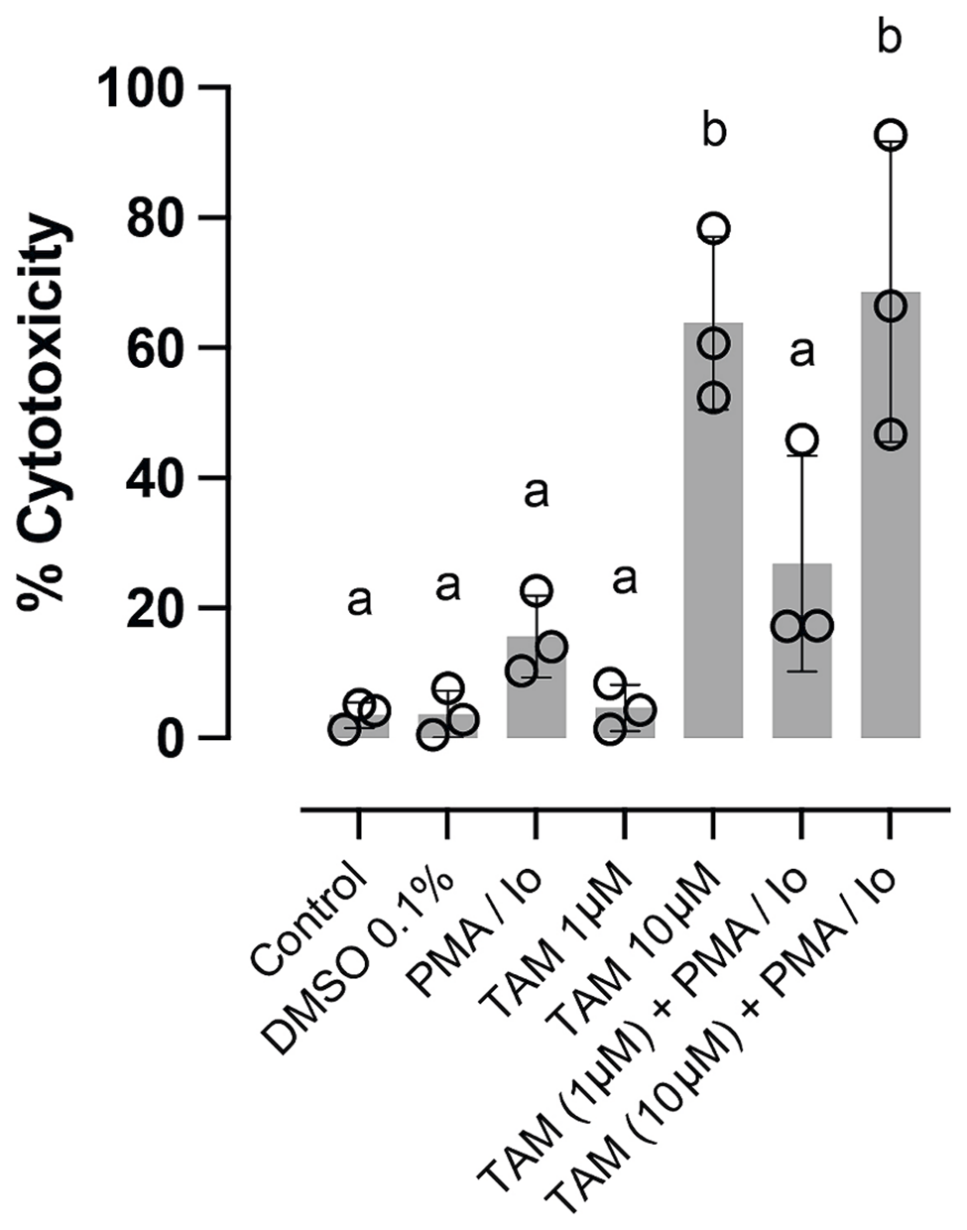
High concentrations of tamoxifen (TAM) induce cytotoxicity in equine PBMC. The proportion of cytotoxicity assessed by lactate dehydrogenase liberation by equine PBMC stimulated or not with PMA (20 ng/mL) + ionomycin (1 μM) and incubated for 6 h with 1 or 10 μM of TAM of its vehicle (DMSO 0.04%). Different letters indicate statistical differences (*p* < 0.05), *n* = 3.

Finally, we evaluated cell viability through a cytotoxicity assay to assess whether the changes induced by TAM at its highest concentration in the expression of specific genes related to the immune response. Figure 3 shows the effect of stimulation with PMA/Io and the effect of TAM in the two highest concentrations used in this study, observing that when used at a concentration of 10 μM, whether in the presence or not of the stimulus, cytotoxicity is significantly increased, even beyond 50%. This result shows that although most of the parameters evaluated were not altered in response to the highest concentration used, the changes observed in the expression of *GATA3*, *IL4*, *IL10* and *CTLA4* due to the cytotoxic effect of TAM cannot be ruled out. However, considering the flow cytometry result, it is likely that TAM differentially impacts lymphocytes and monocytes, the latter being the most affected.

## 4 Discussion

The extensive clinical use of tamoxifen in humans permits the observation of certain effects of the drug on the function of the immune system (3, 26–28). This has been explored in different preclinical models of immune-mediated diseases, where tamoxifen has been shown to have positive effects by controlling inflammation and other pathophysiological consequences associated with loss of tolerance (9–15).

Positive clinical effects of TAM on horses can be partially explained due to their impact on neutrophils (16, 17); little has been explored about the adaptive component of the their immune response. In this sense, PMA and Io are widely used for the evaluation of the phenotype of the immune response, particularly in species where more physiological stimuli are not available, since it will lead to the activation of several intracellular signaling pathways, resulting in T-cell activation and the production of a variety of cytokines (29). The PMA/Io cocktail has been demonstrated to significantly increase the expression of IFN-γ, IL-4, IL-17, and IL-10 on equine PMBCs (30–32). Interestingly, the effect of stimulation on the ratio of IFN-γ/IL-4 cells varies with age; this variation was shown between animals younger than 12 weeks and adult animals (32). As expected, our results show that upon stimulation, there was an overexpression of both the genes associated with the Th1 and Th2 phenotypes. However, despite an increase in the expression of the *IL4* gene, this was not the case for the gene *GATA3*, which, together with Stat6, are the transcription factors that command the Th2 response (33). Something similar has been described in the case of rabbit PBMC stimulated with PMA/Io, which upregulates *TBX21* and *IL4* genes soon after stimulation (4 h) (34). That upregulation was persistent at 24 h after stimulation but with a much lower fold-change. Interestingly, no up-regulation of *GATA3* or *STAT6* genes was detected at 24 h. Something similar was observed with the *IL10* gene, detecting an early upregulation and subsequent downregulation. The negative correlation between *IL4* upregulation and *GATA3* downregulation could be explained because the *IL4* upregulation could be associated with other transcription factors in early stages, such as c-Maf, which has been demonstrated to control the production of IL-4, but no other Th2 cytokines, such as IL-5 or IL-13, for which *GATA3* expression is necessary (35). Additionally, GATA3 expression could be inhibited by the overexpression of CTLA4 since CTLA-4 engagement inhibits Stat6 activation, which is essential for GATA3 up-regulation (36). Regarding Th1-associated genes, both *STAT4* and *TBX21* transcription factors were early upregulated in response to stimulus, and their expression increased even more 24 h after stimulation, which was also associated with the increase in the expression of the *IFNG* gene.

Estrogen participates in a wide range of physiological processes, including the immune response, through the estrogen receptors (ER) signaling pathway. Cancer research has gone deep into evaluating the role of ERs in cancer immunity, the role of SERMs in immunosurveillance, and the effect on immune cell activity, with controversial results described in the literature. Some studies show that TAM induces immune-polarizing side effects (IPSE) in cancer patients, promoting a Th2 bias and impairing therapeutic outcomes by decreasing the CD8^+^/Treg ratio and CD8 and Natural Killer (NK) cell activity (26, 27, 37). It has been pointed out that the effect of TAM on the modulation of NK cell activity would not be mediated by classical ERs (38). Additionally, TAM induced a shift of mouse CD4^+^ cells from a Th1 phenotype toward a Th2 phenotype, modulating the immune response and dampening anti-tumoral immunity but also inflammation in case of autoimmunity (14, 28). In contrast, Babina et al. (9), using a murine model of allergen-induced dermatitis, show that preventive treatment with TAM decreases IL-4 production by splenocytes from treated animals while increasing IFN-γ in response to specific antigen stimulation. They also show that treated animals have lower ear swelling, clinical score, and T lymphocyte infiltration in the skin which would be due to this decrease in the allergic response.

The immunomodulatory effects of SERMs are not limited only to TAM. Ospemifene, an ER agonist/antagonist approved for the treatment of dyspareunia and vaginal dryness in postmenopausal women, has potential new indications as an immune modulator. Kao et al. (39) showed this SERM to induce the expression of Th1 cytokines genes such as *IFNG* and *IL2* while decreasing the proportion of CD4^+^ Foxp3^+^ cells. In contrast, TAM failed to induce overexpression of *IFNG*, proposing Ospemifene as an adjuvant therapy to increase the effectiveness of antigen-specific cancer vaccines. On the other hand, estrogen has been shown to promote the amplification and immunosuppressive capacity of Tregs (40–42), which may be associated with the estrogen-induced upregulation of Foxp3 and PD-1, conferring protection against the development of experimental autoimmune disease models (43, 44). Our results show that low concentrations of TAM *in vitro* do not have an impact on the proportions of Tregs nor gene expression of molecules associated with their function, such as *IL10*, *TGFB1*, *CTLA*, and *PDCD1*, which is similar to what is described in human patients treated with TAM plus a gonadotropin-releasing hormone (GnRH) agonist, showing that CD4^+^ and Tregs number and proportions do not change with the treatment (45). Interestingly, *GATA3* is described as a negative regulator of FOXP3 expression (46), therefore, an increase in the expression of the latter could be expected in response to the stimulus with PMA/Io, However, despite a minor increase in the proportion of CD4^+^ Foxp3^+^ cells, this change was not deemed statistically significant. This observation aligns with the absence of alterations in FOXP3 gene expression in PBMC following exposure to either stimulus or TAM, (data not shown).

Our findings indicate that alterations in the expression of certain genes linked to the immune response in response to TAM treatment are evident only at elevated concentrations. This prompted us to inquire whether this could be attributed to a cytotoxic impact on PBMCs rather than solely being associated with gene expression, and indeed, this hypothesis proved to be accurate. Although there are reports describing that TAM has a protective effect on lymphocytes against the cytotoxic effect in patients under treatment with cyclophosphamide, methotrexate, and 5-fluorouracil (47), the evidence from *in vitro* experiments seems to be different. In this sense, impairment in viability has been described for CD4^+^ cells when concentrations of 10 μM are used, also strongly affecting the proliferation of these cells at even lower concentrations (7.5 μM) (48). The same was reported in the same work for Jurkat cells, observing a greater sensitivity of these cells to TAM with a significant decrease in viability against concentrations above 5 μM. The authors describe the effects of TAM at high concentrations, such as the loss of mitochondrial membrane potential and the increased translocation of phosphatidylserine (PS) in the plasma membrane (early apoptosis). Additionally, they indicate that the inhibition of membrane estrogen receptors (GPR30) was able to reverse TAM-induced autophagy, but not its effect on cell viability, speculating that these off-target mechanisms of TAM could be associated with its inhibitory effect on PCK or directly on the mitochondria. Something similar was described by our group on equine neutrophils since this drug is also capable of inducing a marked loss of mitochondrial membrane potential at similar concentrations, which was associated with caspase-3 activation and cell apoptosis (49). The same occurs in the case of PS translocation, demonstrating a significant increase in the case of neutrophils incubated with TAM at a concentration of 2 μM (21). The translocation of PS is an “eat me” signal for phagocytes, mainly macrophages, inducing an increase in the efferocytosis of apoptotic cells and triggering the process of resolution of inflammation (22).

Although the clinical benefits of using TAM in asthmatic horses have been documented (16, 17), the plasma concentrations achieved after a single dose are notably lower when compared to those observed in women undergoing extensive therapeutic regimens (50, 51). In this sense, while women taking 20 mg of TAM daily may attain micromolar concentrations in their plasma, the plasma concentrations in horses are in proximity to the lower levels examined in our study.

In conclusion, the results of the present study suggest that TAM has a significant impact on the expression of genes related with the Th2 immune response. These changes occur at the highest concentrations of the drug, which is also shown to induce a cytotoxic effect on PBMCs; however, a differential effect between lymphocytes and monocytes cannot be ruled out. It is important to keep in mind that the current work is an initial approach to evaluating the effect of TAM on some players of the adaptive immune response, and additional studies are necessary to figure out the role of the adaptive immune system in the therapeutic effect of TAM observed in horses with inflammation of the airways.

## Data availability statement

The raw data supporting the conclusions of this article will be made available by the authors, without undue reservation.

## Ethics statement

The animal study was approved by Comité Institucional de Cuidado y Uso Animal (CICUA). The study was conducted in accordance with the local legislation and institutional requirements.

## Author contributions

MR: Writing – original draft, Investigation. JQ: Writing – review & editing, Validation, Supervision, Methodology. BC: Writing – review & editing, Methodology, Investigation. GM: Writing – review & editing, Writing – original draft, Funding acquisition, Conceptualization. CH: Writing – review & editing, Writing – original draft, Supervision, Project administration, Methodology, Funding acquisition, Formal analysis, Data curation, Conceptualization.
